# Identification of mega-environments and rice genotypes for general and specific adaptation to saline and alkaline stresses in India

**DOI:** 10.1038/s41598-017-08532-7

**Published:** 2017-08-11

**Authors:** S. L. Krishnamurthy, P. C. Sharma, D. K. Sharma, K. T. Ravikiran, Y. P. Singh, V. K. Mishra, D. Burman, B. Maji, S. Mandal, S. K. Sarangi, R. K. Gautam, P. K. Singh, K. K. Manohara, B. C. Marandi, G. Padmavathi, P. B. Vanve, K. D. Patil, S. Thirumeni, O. P. Verma, A. H. Khan, S. Tiwari, S. Geetha, M. Shakila, R Gill, V. K. Yadav, S. K. B. Roy, M. Prakash, J. Bonifacio, Abdelbagi Ismail, G. B. Gregorio, Rakesh Kumar Singh

**Affiliations:** 1 Central Soil Salinity Research Institute, Karnal, India; 2 Central Soil Salinity Research Institute, Regional Research Station, Lucknow, India; 3 Central Soil Salinity Research Institute, Regional Research Station, Canning Town, India; 4 Central Island Agricultural Research Institute, Port Blair, A & N Islands India; 5 Central Coastal Agricultural Research Institute (CCARI), Ela, Goa India; 6 National Rice Research Institute (NRRI), Cuttack, Odisha India; 7 Indian Institute of Rice Research, Telengana, India; 8 Dr. Balasaheb Sawant Konkan KrishiVidyapeeth, Khar Land, Panvel India; 9 Pandit Jawaharlal Nehru College of Agriculture and Research Institute, Karaikal, India; 10 Narendra Deva University of Agriculture & Technology, Faizabad, Uttar Pradesh India; 11 Rajendra Agricultural University, Samastipur, India; 12 Anbil Dharmalingam Agricultural College and Research Institute, Trichy, India; 13 Punjab Agricultural University, Ludhiana, India; 14 Chandra Shekhar Azad University of Agriculture & Technology, Kanpur, Uttar Pradesh India; 15 Centre for Strategic Studies, Salt Lake City, India; 16 Annamalai University, Chidambaram, Tamil Nadu India; 17 Division of Plant Breeding, IRRI, Philippines; 18 Crop and Environmental Sciences Division, IRRI, Philippines

## Abstract

In the present study, a total of 53 promising salt-tolerant genotypes were tested across 18 salt-affected diverse locations for three years. An attempt was made to identify ideal test locations and mega-environments using GGE biplot analysis. The CSSRI sodic environment was the most discriminating location in individual years as well as over the years and could be used to screen out unstable and salt-sensitive genotypes. Genotypes CSR36, CSR-2K-219, and CSR-2K-262 were found ideal across years. Overall, Genotypes CSR-2K-219, CSR-2K-262, and CSR-2K-242 were found superior and stable among all genotypes with higher mean yields. Different sets of genotypes emerged as winners in saline soils but not in sodic soils; however, Genotype CSR-2K-262 was the only genotype that was best under both saline and alkaline environments over the years. The lack of repeatable associations among locations and repeatable mega-environment groupings indicated the complexity of soil salinity. Hence, a multi-location and multi-year evaluation is indispensable for evaluating the test sites as well as identifying genotypes with consistently specific and wider adaptation to particular agro-climatic zones. The genotypes identified in the present study could be used for commercial cultivation across edaphically challenged areas for sustainable production.

## Introduction

Soil salinity negatively affects agricultural production worldwide. More than 800 million hectares of land globally are salt-affected, including both saline and sodic soils, which represent more than 6% of the world’s total land area^1^. In India, 6.73 million ha are under salt-affected area^2^. Rice is considered salt-sensitive as it is not efficient in controlling the influx of Na^+^ through the roots, leading to a rapid accumulation of toxic concentrations in aboveground parts^3–5^. The problem of salinity has been addressed through better management practices and the introduction of salt-tolerant varieties in affected areas. These high-yielding stress-tolerant rice varieties are expected to provide a yield advantage of about 2 t ha^−1^ on these soils^6^. Thus, genetic improvement of salt tolerance in rice appears to be economically feasible and a promising strategy for maintaining stable rice production globally^7–9^. Initial activities toward such efforts involve testing promising lines bred for salinity tolerance across various salt stress locations to assess their stability and to quantify the effect of genotype × environment (G × E) interactions on the growth and yield of these genotypes. But, studies on evaluating various rice genotypes across different salinity stress locations are quite meager and there is a need to understand the dynamics of cultivar behavior under various stress locations. Moreover, given the same level of salt stress across locations, ambient environmental differences might create variation in the performance of a single variety. Substantial area in India is affected by salt stress; hence, it is imperative to identify certain “hot spots” for evaluating genotypes for their actual value for salt tolerance. Because soil salinity is a much more dynamic state than alkalinity, site variations within saline soils over the years also affect trials considerably in such cases. Therefore, the present study seeks to identify superior cultivars for target regions over the years and to evaluate the possibility of subdividing the target regions into different environmental groups. By this, environments with similar interaction with genotypes can be merged, thus decreasing the cost of conducting field trials without compromising the repeatability of the trials. These objectives can be efficiently realized through the GGE biplot analysis technique.

The GGE biplot methodology consists of a set of biplots from which information regarding genotype and test environment evaluation can be interpreted. A biplot is a scatter plot that approximates and graphically displays a two-way table by both its row and column factors such that relationships among the row factors, relationships among the column factors, and the underlying interactions between row and column factors can be visualized simultaneously^10, 11^. The first application of biplots to agricultural data analysis for model selection was made on data from a performance trial of cotton genotypes^12^. This was followed by the application of biplots for the analysis of genotype by environment tables^13–16^. The biplot tool has become increasingly popular among plant breeders and other agricultural researchers since its use in cultivar evaluation and mega-environment analysis was established^17^. Biplots display both G (genotype main effects) and G × E (genotype × environment interaction) components, which are the two important sources of variation that are relevant to cultivar evaluation and have to be considered simultaneously for appropriate genotype and environment evaluation^18–20^. A “which-won-where” view of biplots can be used to demarcate distinct mega-environments to identify the best-performing genotypes in the corresponding environments^21^. The use of biplots has also been extended to visual analyses of other breeding-related data^22^, host by pathogen interactions^23^, diallel cross tables^24^, microarray data^25^, and QTL by environment interactions^26^. Significant improvement, modifications, and refinements took place in GGE biplot analysis leading to the evolution of different types of GGE biplots such as standard deviation (SD)-based, standard error (SE)-based, and heritability adjusted (HA) biplots. Among these, HA-GGE biplots had been argued to be efficient in evaluating environments and genotypes from multi-location trials (MLTs)^27^. Biplot analysis has also been tried in rice for genotype and environment evaluation^28, 29^ and for line × tester data^30^, etc. But, GGE biplots have not been efficiently used for studies on the performance of genotypes across various salinity stress locations except by Sharifi (2012), who selected the best parents for salinity tolerance-contributing traits based on general combining ability (GCA) and specific combining ability (SCA) estimates depicted on a GGE biplot^31^. Hence, the current study was undertaken to appraise the effect of G × E interaction on the grain yield of 53 promising rice genotypes tested across 18 saline and alkaline stress locations for three consecutive years (2011 to 2013) and analyzed for their value using the HA-GGE biplot technique (hereinafter in text will be denoted as GGE only). The main aim was to group various stress locations into distinct mega-environments so that locations with a similar effect on the genotypes could be discontinued for testing in the future and to identify the best locations for future testing, and to ascertain the stable and superior genotypes that could be used as varieties or suitable donors for a particular mega-environment or for wider adaptation across salt stress environments.

## Results

### Analysis of variance

Analysis of variance was done for the yield data from individual years (2011, 2012, and 2013) and three-year (2011–13) combinations (Tables 1 and 2). The results indicated that genotype, location, and genotype × location effects were significant in all three years (2011, 2012, and 2013) as well as in combined analysis for three years (2011–13). Additionally, the relative contribution of each source of variation to the total sum of squares was calculated for comparison.

**Table 1:** ANOVA and basic statistics in individual years (2011, 2012, and 2013).

Source	2011		Percent contribution to total SS	2012		Percent contribution to total SS	2013		Percent contribution to total SS
	df	MS		df	MS		df	MS	
Genotype (G)	24	4251672**	7.35	29	5828938**	6.62	30	4089298**	4.4
Location (L)	11	54308145**	43.05	13	1.3E + 08**	66.41	16	1.13E + 08**	65.4
G × L	251	2454228**	44.39	362	1542148**	21.86	440	1393591**	22.1
Blocking	24	321024	0.56	28	604290.4	0.66	34	478228.4	0.6
Error	541	119245.2		781	145545		929	222628.7	
Grand mean (kg ha^−1^)		1904.917			2385.858			2649.604	
SE (kg ha^−1^)		345.319			381.5035			471.8354	
LSD_0.05_ (kg ha^−1^)		565.1532			624.3732			772.2114	
CV (%)		18.13			15.99			17.81	
H (V_g_/V_p_)		0.42			0.74			0.66	

df = degrees of freedom; MS = mean square; SS = sum of squares; ** = significant at 1% level of probability.

**Table 2:** ANOVA and basic statistics across three years (2011–13).

Source	2011–13		
	Df	MS	Percent contribution to total SS
Genotype (G)	12	4876627**	1.64
Year (Y)	2	61830663**	3.46
Location (L)	17	85264239**	40.52
L × Y	23	36226572**	23.29
Block (L × Y)	86	353283.8**	0.85
G × Y	24	1929503**	1.29
G × L	200	2348058**	13.13
G × L × Y	273	1483738**	11.32
Error	1011	158898.4	
Grand mean (kg ha^−1^)		2549.09	
SE (kg ha^−1^)		398.62	
LSD_0.05_ I (kg ha^−1^)		653.77	
LSD_0.05_ II (kg ha^−1^)		856.99	
CV (%)		15.64	
H (V_g_/V_p_)		0.43	

Df = degrees of freedom; MS = mean square; SS = sum of squares; **− = Significant at 1% level of probability.

Genotype by location (G × L) contributed the most (44.39%) to the total variation, closely followed by location (43.05%), during 2011. The effect of location remained highest (66.41% and 65.40%) in 2012 and 2013, respectively. Genotypes had greater interaction effects with locations or locations by year but the per se contribution was observed to be 7.35%, 6.62%, and 4.4% in 2011, 2012, and 2013, respectively, which was quite less comparatively. We noticed a profound effect of location (40.52%) in 2011–13. The main effects (G and Y) and genotype by year (G × Y) showed smaller effects than other sources of variation. Overall, the impact of different factors on yield can be ordered as Location (L) > Location × Year > Genotype × Location > Genotype × Location × Year > Year > Genotype > Genotype × Year. The different summary statistical parameters, mean, standard error, and standard deviation were estimated in different years (Supplementary Table 1).

### Evaluation of test environments

**Figure 1:**
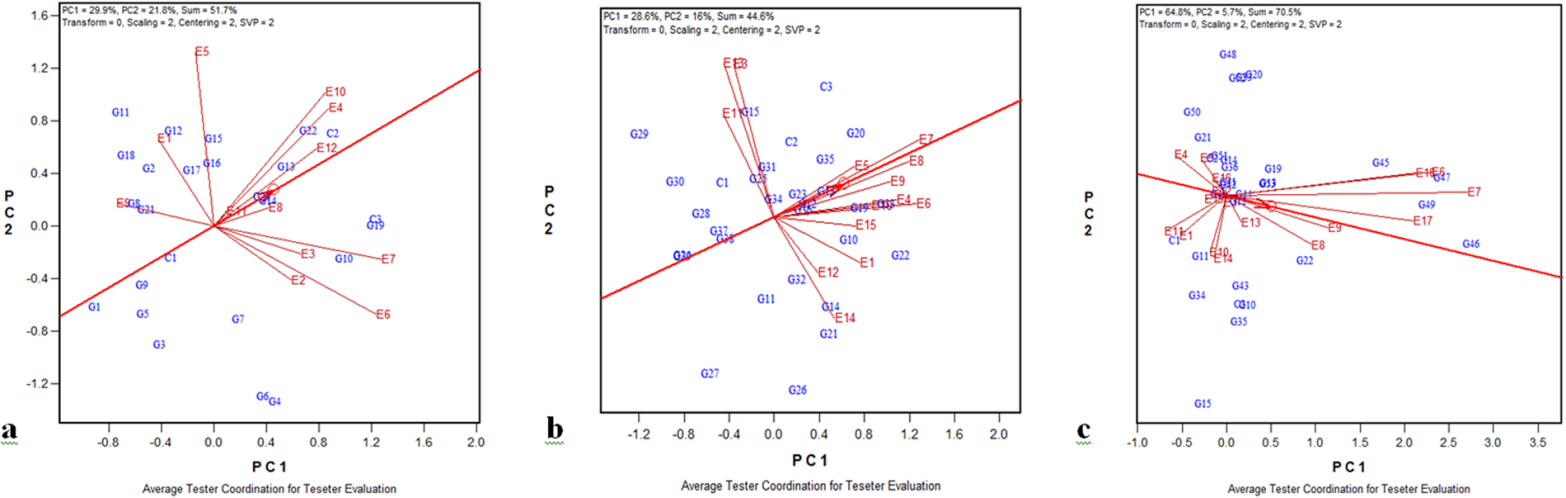
Association among the test environments and distance from the ideal genotype, based on the average environmental coordinate (AEC), considering stability and adaptability of rice genotypes evaluated across saline and alkaline environments for grain yield in (**a**) 2011, (**b**) 2012, and (**c**) 2013 growing seasons.

The biplot explained 51.7%, 44.6%, and 70.5% of the total variation of the environments in 2011, 2012, and 2013, respectively. GGE biplot showed two distinct clusters in 2011: one containing BSKKV, Kharland RS, Panvel (E10); CCARI, Goa (E4); and ADACRI, Trichy (E12), and the other containing IIRR, Machilipatnam (E3); CSSRI, Karnal sodic environment (E7); NRRI, Cuttack (E2); and CSSRI, Karnal saline environment (E6) (Fig. 1a). The closest association was observed between the environments BSKKV, Kharland RS, Panvel (E10), and CCARI, Goa (E4). The location NDUAT, Faizabad (E9) showed negative or no correlation with most of the locations (except CIARI, Port Blair (E1) and PAJANCOA, Karaikal, Puducherry (E5)) and hence can be considered distinct. CSSRI, Karnal saline environment (E6) had the longest vector and hence was a highly discriminating location (Fig. 1a). But, its angle with the AEA is greater and hence less representative of other environments. The location ADACRI, Trichy (E12) was highly representative of other locations. Considering the above two qualities together, ADACRI, Trichy (E12) was the ideal location for testing genotypes for salt tolerance with its appreciable discriminating ability and representativeness (Supplementary Table 2). RAU, Pusa, Bihar (E11) showed the least discriminating ability and the locations NDUAT, Faizabad (E9) and CIARI, Port Blair (E1) were the least representative of other locations. There were three clusters of environments in 2012: CSSRI RRS, Canning Town (E13); IIRR, Machilipatnam (E3); and RAU, Pusa, Bihar (E11). Among these three, E13 and E3 were closely associated (Fig. 1b). The second cluster consisted of ADACRI, Trichy (E12); CSS, Kolkata (E14); and CIARI, Port Blair (E1), and the other contained the remaining environments. CSSRI Karnal sodic environment (E7) had the longest vector and hence was highly discriminating. The locations CSSRI RRS, Lucknow (E8); CSSRI Karnal sodic environment (E7); and NDUAT, Faizabad were highly representative environments (Fig. 1b). The remaining locations displayed medium to high discriminating power in 2012. Overall, the location CSSRI Karnal sodic environment (E7) can be considered ideal for evaluating genotypes. RAU, Pusa, Bihar (E11) was the least consistent (representative) location in 2012. GGE biplot showed four clusters in 2013 (Fig. 1c). The first cluster contained coastal locations such as CCARI, Goa (E4) and PAJANCOA, Karaikal, Puducherry (E5); the second contained mostly inland saline and alkaline locations – CSSRI, Karnal saline environment (E6); CSSRI, Karnal sodic environment (E7); CSSRI RRS, Lucknow (E8); NDUAT, Faizabad (E9); PAU, Ludhiana (17); and CSSRI, Nain Farm, Panipat (E18). Among these, CSSRI, Karnal saline environment (E6) was more associated with CSSRI, Nain Farm, Panipat (E18), and CSSRI RRS, Lucknow (E8) with NDUAT, Faizabad (E9). The third cluster contained RAU, Pusa, Bihar (E11); CIARI, Port Blair (E1); BSKKV, Kharland RS, Panvel (E10); and CSS, Kolkata (E14). Among these four locations, the first two and the last two were more closely associated. The remaining environments were in the fourth and final cluster. CSSRI, Karnal sodic environment (E7) repeated the same performance as in 2012 as it had the longest vector, making it more discriminating than other environments, and the environment NDUAT, Faizabad (E9) showed a smaller angle with the AEA and hence was a highly representative environment (Fig. 1c) in 2013.

The discriminating ability, representativeness estimates, and desirability index of different locations were pooled and the mean values are presented in Table 3. The locations CSSRI, experimental farm, Nain (E18) and PAU, Ludhiana (E17) showed high discriminating power and come under the most desirable locations. But, these locations were included in the trial only in 2013 and are based on a single year’s values; hence, these could be downplayed for now. Besides these two locations, Karnal sodic environment (E7; 1.82) showed the highest discriminating ability, followed by Karnal saline environment (E6; 1.67), and carries the highest desirability index value based on three years of data. Desirability index is the overall manifestation of pooled performance based on the discriminatory power of a location and representativeness; therefore, it could be a better parameter to identify better locations for experimental setup. Other relatively desirable locations are CSSRI RRS, Lucknow (E8), NDUAT, Faizabad (E9), BSKKV, Kharland RS, Panvel (E10), and CCARI, Goa (E4). The location CSAUT, Kanpur had weak discriminating ability (E16; 0.24). The remaining locations possessed appreciable discriminating ability, with values ranging from 0.58 (ADACRI, Trichy, E12) to 0.94 (BSKKV, Kharland RS, Panvel, E10, and CSSRI RRS, Lucknow, E8). Similar to discriminatory power, PAU, Ludhiana (E17) and CSSRI, experimental farm, Nain (E18) showed high representativeness but again based on a single year (2013) of data; however, CSSRI RRS, Lucknow (E8; 0.98) and saline and sodic/alkaline microplots in Karnal (E6 and E7) showed high representativeness compared with all locations (based on three years). Many environments did not appear as good locations due to low desirability index (CIARI, Port Blair (E1), NRRI, Cuttack (E2), IIRR, Machilipatnam (E3), RAU, Pusa, Bihar (E11), CSSRI RRS, Canning Town (E13), and CSAUAT, Kanpur (E16)) and most of them were saline environments.

**Table 3:** Standardized test location evaluation parameters.

Location	Discriminating power Mean ± SD	Representativeness Mean ± SD	Desirability index Mean ± SD
E1	0.77 ± 0.09	0.00 ± 0.63	0.10 ± 0.57
E2	0.73 ± 0.00	0.41 ± 0.00	0.15 ± 0.00
E3	0.70 ± 0.62	0.23 ± 0.39	0.14 ± 0.35
E4	1.03 ± 0.27	0.34 ± 1.08	0.50 ± 1.03
E5	0.89 ± 0.41	0.22 ± 0.89	0.34 ± 0.65
E6	1.67 ± 0.57	0.80 ± 0.25	1.35 ± 0.63
E7	1.82 ± 0.78	0.90 ± 0.14	1.67 ± 0.85
E8	0.94 ± 0.44	0.98 ± 0.02	0.94 ± 0.49
E9	1.01 ± 0.23	0.42 ± 1.00	0.61 ± 1.02
E10	0.94 ± 0.35	0.61 ± 0.59	0.75 ± 0.68
E11	0.63 ± 0.43	0.05 ± 0.86	0.01 ± 0.50
E12	0.58 ± 0.41	0.10 ± 1.01	0.34 ± 0.55
E13	0.84 ± 0.70	0.38 ± 0.43	0.04 ± 0.31
E14	0.84 ± 0.22	0.09 ± 0.10	0.22 ± 0.07
E15	0.49 ± 0.35	−0.03 ± 1.28	0.25 ± 0.57
E16	0.24 ± 0.00	−0.80 ± 0.00	−0.14 ± 0.00
E17	2.12 ± 0.00	1.00 ± 0.00	2.16 ± 0.00
E18	2.15 ± 0.00	0.94 ± 0.00	2.04 ± 0.00

### Performance and stability of rice genotypes across salinity and alkalinity stresses

**Figure 2:**
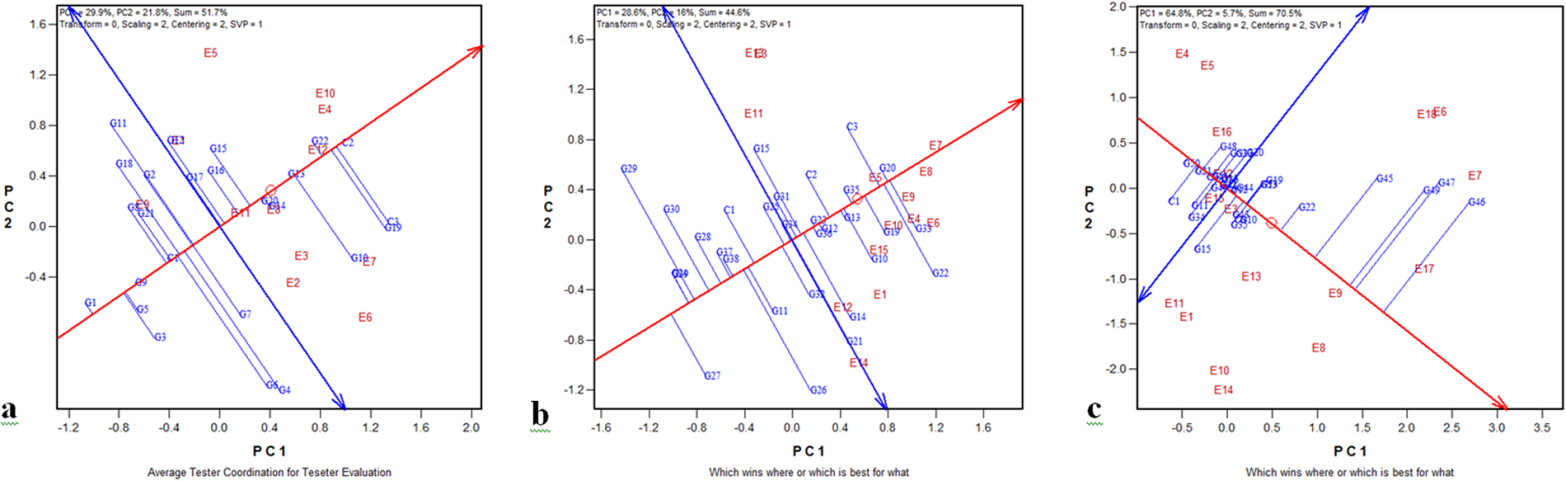
GGE biplot of mean and stability of rice genotypes for yield and specific genotype × environment interactions in (**a**) 2011, (**b**) 2012, and (**c**) 2013.

Within a single mega-environment, genotypes should be evaluated on both mean performance and stability across environments. The average-environment coordination (AEC) views of the GGE biplot for grain yield for 2011, 2012, and 2013 are shown in Fig. 2a–c. CSR 27 (C2) recorded the highest average grain yield in 2011 (Fig. 2a). CSR 27 (C2), CSR-2K-262 (G22), NDRK 11-4 (G13), CSR-2K-242 (G20), and NDRK 11-5 (G14) were the most stable genotypes with above-average yields. Thus, the check CSR 27 was the most ideal genotype with the highest mean yield and stability; however, CSR-2K-262 (G22) was the most ideal genotype among the tested genotypes. The genotype PNL 9-1-2-7-4-6-1 (G23) was the most stable genotype with above-average yield in 2012 (Fig. 2b). CSR 12-B 23 (G33) recorded the highest mean yield but genotype CSR-2K-242 (G20) was the ideal genotype with high stability and grain yield. Other stable genotypes with above-average yields were NDRK 11-7 (G35), NDRK 11-4 (E13), NDRK 11-3 (G12), and PNL 4-35-20-4-1-4 (G36). CSRC(D)12-8-12 (G46) recorded the highest average grain yield in 2013 but its performance was found highly variable across environments (Fig. 2c). NDRK 11-3 (G12) was the most stable genotype, but with near average yield. Unfortunately, no genotype was found ideal in this year, although CSR-2K-262 (G22) was found comparatively more stable and an above-average yielder than the other genotypes (Table 4).

**Table 4:** Summary of ideal genotypes and genotypes with stable and high mean yields in different seasons (2011–13).

**Year**	**Ideal/near-ideal genotype**	**Stable genotype**	**High mean**
2011	CSR 27 (C2)	CSR 27 (C2), NDRK 11-4 (G13), CSR-2K-242 (G20)	CSR 27 (C2), CSR-2K-262 (G22)
2012	CSR-2K-242 (G20)	PNL 9-1-2-7-4-6-1 (G23), NDRK 11-3 (G12), NDRK 11-7 (G35)	CSR 12-B 23(G33), CSR-2K-242 (G20)
2013	CSR-2K-262 (G22)	NDRK 11-3 (G12), NDRK 11-1 (G10), NDRK 11-5 (G14)	CSRC(D)12-8-12 (G46)
2011–13	CSR-2K-219 (G19)	CSR-2K-219 (G19), NDRK 11-1 (G10), RP4353-MSC-38-43-6-2-4-3 (G14)	CSR-2K-262 (G22)

### Identification of which-won-where and mega-environment

One of the most attractive features of a GGE biplot is its ability to show the which-won-where pattern of a genotype by environment dataset. This plot consists of a polygon with perpendicular lines, called equality lines, drawn onto its sides. These lines divide the polygon into various sectors. Genotypes located on the vertices of the polygon are the best performers in one or more environments falling within a particular sector.

**Figure 3:**
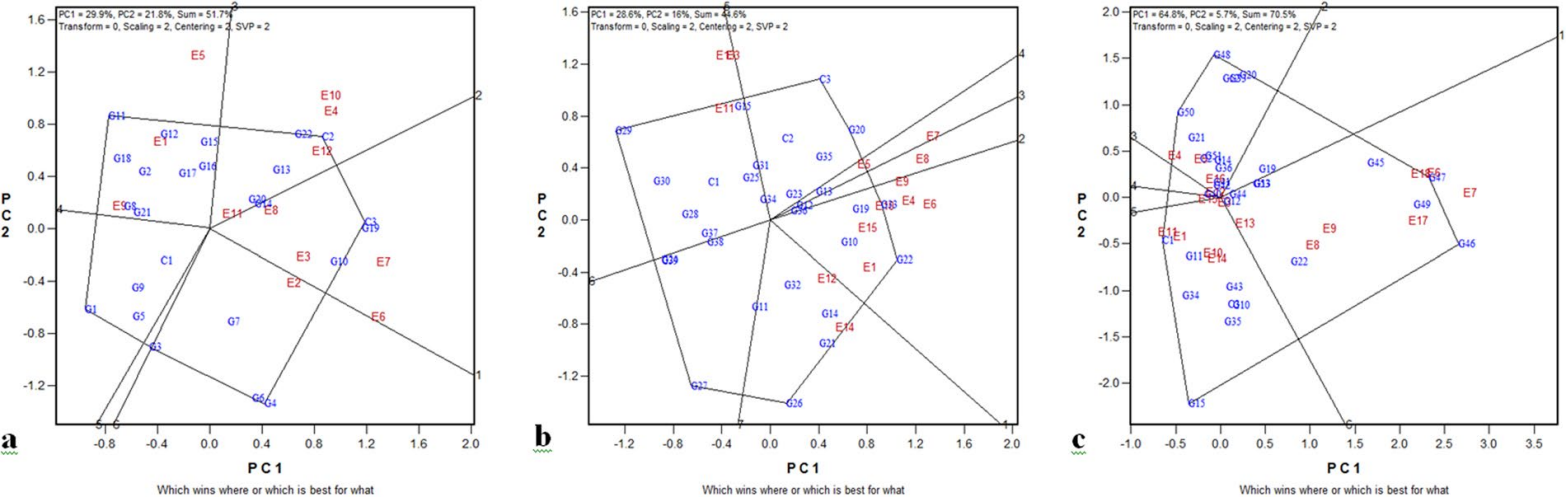
GGE biplot identification of winning genotypes and their related mega-environments in (**a**) 2011, (**b**) 2012, and (**c**) 2013.

The biplot showed four sectors containing all the test environments in 2011 and accordingly four mega-environments were identified (Fig. 3a): one mega-environment had four locations, IIRR, Hyderabad (E3); CSSRI, Karnal saline environment (E6); CSSRI, Karnal sodic environment (E7); and CSSRI RRS, Lucknow (E8); the second consisting of CCARI, Goa (E4); BSKKV, Kharland RS, Panvel (E10); RAU, Pusa, Bihar (E11); and ADACRI, Trichy (E12); the third had three locations, CIARI, Port Blair (E1); PAJANCOA, Karaikal, Puducherry (E5); and NDUAT, Faizabad (E9); and the fourth had only one location, NRRI, Cuttack (E2); hence, it would not likely be called a mega-environment. CSR 36 (C3) and CSR-2K-219 (G19) were the winning genotypes in the first mega-environment, CSR 27 (C2) was the winner in the second, and NDRK 11-2 (G11) was the winning genotype in the third mega-environment (Supplementary Table 3).

In 2012, the biplot grouped the test locations into three mega-environments (Fig. 3b). The first mega-environment had three locations, IIRR, Machilipatnam (E3); CSSRI RRS, Canning Town (E13); and RAU, Pusa, Bihar (E11); the second had six locations, CSSRI, Karnal saline environment (E6); BSKKV, Kharland RS, Panvel (E10); CIARI, Port Blair (E1); Annamalai University (E15); CCARI, Goa (E4); and NDUAT, Faizabad (E9); the third contained only two locations, ADACRI, Trichy (E12) and CSS, Kolkata (E14). Amalmana (G29) was the winning genotype in the first mega-environment while CSR-2K-262 (G22) was the winner in the second and CR 2815-4-3-1-1-1-1 (G26) was the winner in the last mega-environment.

The biplot was divided into three mega-environments in 2013 (Fig. 3c). The first mega-environment had seven locations, CSSRI, Karnal saline environment (E6); CSSRI, Karnal sodic environment (E7); CSSRI RRS, Lucknow (E8); NDUAT, Faizabad (E9); CSSRI RRS, Canning Town (E13); PAU, Ludhiana (E17); and CSSRI, Nain Farm, Panipat (E18), with CSRC(D)12-8-12 (G46) and CSRC(D)13-16-9 (G47) being the winning genotypes. The second mega-environment had four locations, ADACRI, Trichy (E12); CSAUAT, Kanpur (E16); PAJANCOA, Karaikal, Puducherry (E5); and CCARI, Goa (E4), and CR 2814-2-4-3-1-1-1 (G48) and CR 2815-4-23-7-5-2-1-1 (G50) were the winners in this mega-environment. The third mega-environment consisted of six locations, CIARI, Port Blair (E1); RAU, Pusa, Bihar (E11); PAJANCOA, Kharland RS, Panvel (E10); IIRR, Machilipatnam (E3); Annamalai University TN (E15); and CSS, Kolkata (E14), with CST 7-1 (C1) and RP 4353-MSC-38-43-6-2-4-3 (G15) as the winning genotypes.

**Figure 4:**
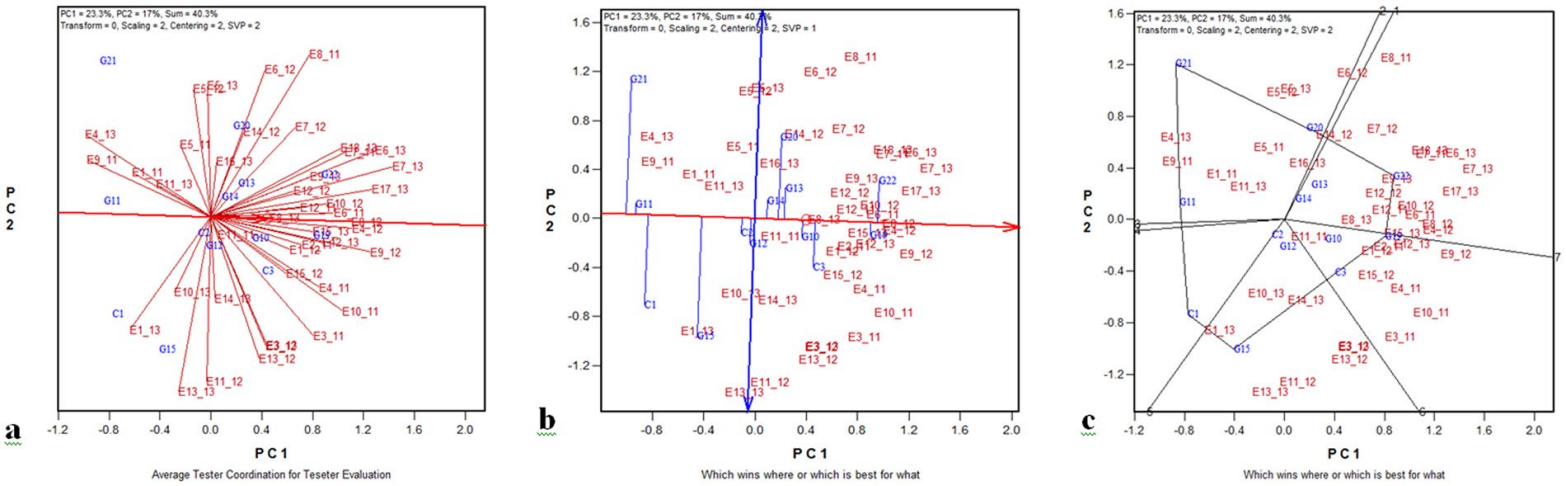
(**a**) Environment, (**b**) genotype, (**c**) and “which-won-where” of GGE biplots constructed using common genotypes tested across three years (2011–13).

The pooled data based on 13 common genotypes across three years over all the tested environments were analyzed and plotted for graphical representation of average environmental coordinate (AEC) and similarity of test locations, mean and stability of genotypes, and “which-won-where” for grain yield (Fig. 4a–c). The biplot explained 40.3% of the GE interaction variance. CSSRI, Karnal sodic environment (E7_13), followed by CSSRI RRS, Lucknow (E8_11), was the most discriminating location (Fig. 4a). The location CSSRI RRS, Lucknow (E8_12 and E8_13) was found highly representative. The location NDUAT, Faizabad (E9_12) was a better location than all other locations based on ideal environment and discrimination power. CSR-2K-262 (G22) showed the highest mean grain yield and CSR-2K-219 (G19) was considered as the most stable relatively (Fig. 4b). NDRK 11-1 (G10), NDRK 11-4 (G4), NDRK 11-5 (G5), and CSR 36 (C3) were other stable genotypes with above-average yields. From the “which-won-where” graphical view, CSR-2K-262 (G22) was the best and most adapted genotype across 16 year/location combinations (Supplementary Table 3 and Fig. 4c). Genotype CSR-2K-255 (G21) was the winning genotype across 10 year and location combinations (Fig. 4c). Yield data of the ten best-performing genotypes in the individual years were pooled across salinity- and alkalinity-affected locations separately to isolate genotypes suitable for salinity and alkalinity stress conditions (Supplementary Table 4). Genotypes CSR-2K-262, RP 4353-MSC-38-43-6-2-4-3, CSR-2K-219, and NDRK 11-1 were found superior under saline conditions and genotype CSR-2K-262 was the only genotype that fell into the top 10 consistently over three years.

## Discussion

Plant breeders routinely conduct multi-location trials over years to evaluate the performance of test entries and this provides a robust basis for inferences and recommendations for varietal adoption and release for commercialization. The total sum of squares for the data obtained from such trials can be partitioned into assignable and non-assignable (residual) causes, where assignable causes are from three main sources: the genotype main effect, the environment main effect, and genotype × environment (G × E) interaction. It is the third component that makes the evaluation of genotypes complicated because of the differential performance of cultivars in different locations. Understanding of the degree and causes of G × E interaction is highly useful for framing breeding objectives, identifying ideal test locations, and formulating varietal release recommendations^32^. It is also very important for evaluating the adaptability and stability of the genotypes. There are numerous methods by which this G × E interaction can be quantified and understood. Analysis of variance (ANOVA), principal component analysis (PCA), and linear regression models are traditionally applied to analyze MLT data^33^. More improved methods such as additive main effects and multiplicative interactions (AMMI)^14^ and genotype main effect plus genotype × environment interaction (GGE) biplot^17, 20^ are being preferred for more meaningful inferences as they combine the power of additive and multiplicative models. AMMI has limited applications because it gives AMMI stability value (ASV), but breeders need more than only high stability; they also need higher yield^33^. The GGE biplot model efficiently analyzes multi-location data and displays the analyzed information as an easily interpretable graphic illustration. There are different types of GGE biplots depending on the type of scaling used before the singular value partitioning – without scaling, scaling by standard deviation of genotype means within environments (*SD*-Scaled), scaling by the standard error within the environment (*SE*-Scaled), and heritability adjusted GGE (HA-GGE) biplot^17, 27^. In HA-GGE biplot, the vector length of testers (environments) is proportional to the square root of heritability in the environments^27^. It is argued that the HA-GGE biplot is the most appropriate among all biplots for both environment and genotype evaluation^27^; hence, we used Heritability Adjusted GGE biplot in the present study.

A number of studies^34–40^ exist on GGE analyses in many crops such as rice, cotton, sorghum, and sugarcane, mostly for non-stress environments, but none of them covered across salt stress-affected locations in any crop. Several studies aimed to look for the highest-yielding stable genotypes and mega-environments in the respective regions in northern Bangladesh^41^, Brazil^42^, and Nigeria^28^. This technique was even employed to study disease reactions^43^ and combining ability data^30^. We could find only one paper from Sharifi (2012) that applied GGE biplot by analyzing six parents full- diallel progeny to identify the best parents, Sepidrod and IRFAON-215, to be used for breeding programs contributing to salinity tolerance^31^. Few AMMI analyses were previously attempted to identify stable genotypes tested across different salinity stress locations^34, 44, 45^, but the present study is the first to employ HA-GGE biplot to analyze the large dataset from multi-location trials carried out across different salinity and alkalinity stress locations in India over three years.

Soil salinity is a relatively dynamic state, both temporally and spatially^46^, whereas alkalinity is relatively less dynamic but still greatly affected by weather conditions. The significantly higher proportion of the source of variation due to L followed by G × L for individual years or in combined analysis is as expected. A significant contribution of location effect was previously reported in many studies^37–40^. Genotypic effects were also highly significant and their interplay with L is of utmost importance from the breeding point of view. Plant breeders always look for genotypes that perform better across locations with minimum G × E interaction, but that seldom happens, especially under soil stress environments. GGE biplots facilitate discerning stable and ideal genotypes and ideal environments from genotype and environment views, respectively. The “ideal” test environment is a virtual environment that has the longest vector of all test environments (most discriminating) and it is located on the AEC abscissa (most representative)^32^. Because of temporal and spatial differences, we did not find repeatability for the ideal environment over the years; rather, different environments stood out as ideal in different years, such as ADACRI, Trichy (E12) in 2011; CSSRI, Karnal sodic environment (E7) in 2012; and PAU, Ludhiana (E17) in 2013. When all three years are considered together, the location CSSRI, Karnal sodic environment (E7) turned out to be the most ideal location with significant discriminating ability and consistency. Different ideal environments within the study locations over the years are quite obvious given the highest contribution of location to the total variance. Genotype performance is dependent on these locations that suit specific genotypes with stable salinity/alkalinity tolerance across various locations. Since alkalinity is less dynamic than salinity, the Karnal sodic/alkaline environment (E7), which is quite controlled and stressful, was found highly discriminating across the years (2012, 2013, and 2011–13). This is further supported by the estimates of discriminating ability averaged over the years. Hence, this location is capable of revealing true differences among genotypes and could be used for culling out inferior (sensitive) and unstable genotypes.

Another important application of GGE biplot is to identify and segregate less appropriate test sites. Such locations could be dispensed with if resources are scarce^47^. Although a couple of such sites were identified in the present study, it would be wise to continue such multi-location testing for a few more years before dropping less effective sites, considering the huge temporal variability. This is evident from the results that no environment appeared twice in consecutive years as a non-discriminating one. For example, RAU, Pusa, Bihar (E11) (in 2011) and IIRR, Machilipatnam (E3), ADACRI, Trichy (E12), Annamalai University (E15), and CSAUAT, Kanpur (E16) (in 2013) were weak discriminating locations. These locations can be intuitively considered ineffective as the true performance of genotypes was masked considerably by the environmental effect in the respective years. The second category could be to identify those locations that were less discriminating comparatively such as CSSRI, RRS, Lucknow (E8), NRRI, Cuttack (E2), and IIRR, Machilipatnam (E3) in 2011; ADACRI, Trichy (E12) and Annamalai University (E15) in 2012; and many (CIARI, Port Blair (E1), CCARI, Goa (E4), PAJANCOA, Puducherry (E5), BSKKV, Kharland RS, Panvel (E10), RAU, Pusa, Bihar (E11), CSSRI, RRS, Canning Town (E13), and CSS, Kolkata (E14)) in 2013. Such variation for the discriminating ability of the locations over years suggests that decisions on continuing or dropping a location should be made only after series of tests over years, not based on two or three years of data, as a short period of information about locations could mislead the inferences^27^. Locations such as ADACRI, Trichy (E12), which was not highly discriminative but was representative in 2011, turned into a non-representative location with moderate discriminating ability in 2012. The same location in 2013 was neither discriminating nor representative. Contrary to this example, the CSSRI, Karnal saline microplots (E6) were non-representative of the ideal environment in 2011 but were less discriminative but more representative in 2012, but were found to be a near-to-representative and better discriminative environment.

This is also evident from the desirability index. One year of data showed the highest desirability for PAU, Ludhiana (E17) and CSSRI, Nain Farm (E18), but there is always risk in drawing inferences from one year of data. Desirability index based on three years clearly shows those environments as most desirable that have enough stress to segregate the tolerant and sensitive genotypes. CSSRI saline and alkaline microplots (E6 and E7) are the controlled facilities where there is minimal chance for any genotype to escape from stress. Moreover, saline environments are relatively more dynamic for the degree of stress; therefore, most of the saline sites had low or poor desirability index. Hence, one should not discontinue trials at certain locations based on a few years of data. Before making a decision about locations, especially under a dynamic state of soil conditions in salt-affected environments, we suggest considering about 5 years of data for decisions on omitting a trial site. Similar prioritization for targeting the most representative and discriminative locations has been done by other studies also^17, 35, 39, 40, 48^.

A genotype is considered ideal if it has high mean yield and is less variable across locations and seasons. Therefore, genotypes located closer to the virtual “ideal genotype” are more desirable. In the present study, different genotypes were found ideal in different years. This is mainly because not all the genotypes over years were the same and some of the poor performers were dropped over years. The quality of the data over years could be considered quite reliable due to considerable CV (16–18%) and moderate to high broad-sense heritability (42–74%) over years (Table 1). The CV invariably is very high under stress environments where many of the genotypes could die during the course of evaluation; that’s why even very high CV is admissible for such experiments. Since every year the data comprised about 15 location results, the CV over the location on pooled analysis for individual years came down, which is quite obvious. Individual environmental data analysis from highly stressful sites such as CSSRI saline (E7) and alkaline (E8) microplots, where many of the entries could not survive or reach maturity, showed the CV to be very high as expected, and this is widely accepted in stress environments as long as reliable heritability estimates are obtained. The same was true with pooled analysis done over the years (Table 2). The dynamic state of salt stress over years further confounds the individual genotype performance. This is a bit different than in other studies^21, 29^ because of the high soil heterogeneity among locations as well as variation over the years. As breeders, we like to develop a highly adaptive genotype that is good over environments and years but, for the regional perspective, this is not practical in the long term just to avoid the buildup of virulent races that can devastate large areas such as the infamous devastation of maize crops in the United States in the late 1960s and early 1970s because of *Bipolaris maydis* race T (formerly known as *Helminthosporium maydis*) disease on maize hybrids^49^. The genotypes CSR 27 (C2) and CSR-2K-262 (G22) (in 2011), CSR-2K-242 (G20) and NDRK 11-7 (G35) (in 2012), and CSR-2K-219 (G19) and NDRK11-1 (G10) (in 2011–13) were found ideal or near ideal in the present study (Table 4). Variety CSR 27 is released as a national variety for salt tolerance in India and again was shown to be ideal in salt stress locations. Although there was not much commonality among the genotypes identified as stable or near ideal, the data suggest that CSR-2K-219 (G19), CSR-2K-242 (G20), and CSR-2K-262 (G22) appeared again and again as stable, ideal, or top yielders in different environments and also over years. This implies that these genotypes seem to be most promising despite the high soil heterogeneity prevailing at the salt-affected test locations. Indeed, salt stress could be such a dynamic state in which the degree of stress experienced by a genotype can vary every day depending upon the soil moisture, temperature, and relative humidity of the microclimate^50, 51^. The environments used in the current study represent saline and alkaline soils (different because of the chemical composition of salts) and also coastal and inland salinity (different because of the vicinity of the seacoast and mode of salinization). Hence, the genotypes as given in Table 4 could be considered suitable for such soils. When three years of data were considered together, CSR-2K-219 (G19) and CSR-2K-262 (G22) were found promising over years (2011–13). Breeders need not only stable varieties; these should also have higher yields to make them attractive to farmers for adoption. It is important to consider here that the checks continue to outperform test genotypes in many instances. CSR 27 (C2) was the ideal genotype in 2011 and CSR 36 (C3) in 2011–12 (combined analysis of 2011 and 2012, not shown). These two were the winning genotypes in two mega-environments in 2011 as well. Considering these results, CSR-2K-219 and CSR-2K-262 are the most suitable genotypes for the majority of the salt stress locations, followed by CSR-2K-242. These genotypes were superior among all others with higher mean yields and stability; thus, they could be nominated for the formal release process and commercialization. A number of studies, although not exactly on GGE, looked for stable rice genotypes across salt stress locations and nominated the most stable and highest-yielding genotypes in the national varietal testing process for release^34, 44, 48, 52^.

As mentioned earlier, salinity and alkalinity (or sodicity) are two different kinds of salt stress although both exert stress due to high Na^+^ concentration. Na^+^ is in soil solution as soluble salt in saline soils but the same Na^+^ is adhered on clay micelles through a colloidal complex in alkaline soils^5^. In this study, genotype performances were compared across salinity- and alkalinity-affected locations separately. Many genotypes that respond well under salinity are also tolerant of alkalinity. This could be quite common but not a rule of thumb^5, 50, 51^. Some genotypes performed well under salinity but not under alkalinity and vice-versa and some performed well under both stresses. This implies that the genotypes showed specific adaptation to saline and alkaline conditions by virtue of different combinations of mechanisms/genes governing their tolerance^53–55^. The top ten genotypes in each year were separated and the genotypes that were consistently high yielding across three years were shortlisted. Genotypes CSR-2K-262, RP 4353-MSC-38-43-6-2-4-3, CSR-2K-219, and NDRK 11-1 were found to be consistently superior under saline conditions and, interestingly, CSR-2K-262 was the only genotype that fell into the top ten in three years consistently under alkaline/sodic conditions also. Thus, CSR-2K-262 could be considered as suitable for both saline and sodic conditions and could be considered as one of the best findings. Based on results over years, in 2016, CSR-2K-262 was released as rice variety CSR46 for the salt-affected areas of provinces of Uttar Pradesh in India.

Dividing a target region into meaningful sub-regions is the only way to make use of any repeatable genotype by location interactions in plant breeding. Using a maize (*Zea mays* L.) multi-environment trial (MET) dataset, Gauch and Zobel (1997) presented a “which-won-where” methodology for identifying mega-environments^21^. Data from multiple years are needed for such groupings. Attempts to group test locations into mega-environments using multi-seasonal data were previously reported in different crops, for example, in spring wheat^56^, cotton^57^, sugarcane^40^, and rice^47^. As of today, there are no reports on grouping rice-growing salt stress-affected locations of India into different zones. This is highly essential for deploying zone-specific rice genotypes for the management of salt-affected soil in India. “Which-won-where” plots constructed in the present study grouped the locations used for testing each year into different mega-environments: three mega-environments in 2011, three in 2012, and three in 2013 were identified. But, the grouping did not correspond with the geographic location or stress type (salinity and alkalinity) prevalent at the locations. However, a higher degree of stress (E6, E7, E11, and E12) always found space in one or another sub-group while some of the environments with less stress during the growing season (E2 and E17) could not find space in the mega-groups. Some of the non-repeatability of the “which-won-where” pattern could be because new locations and new genotypes were added every year from 2012 and 2013 and this has been reported earlier^56^ (details were given in Materials and Methods). This might have caused yearly fluctuations in genotypic and environmental scores, thereby disturbing the “which-won-where” pattern. As pointed out by Gauch and Zobel (1997), if the which-win-where pattern is used as the sole criterion, the identified mega-environments would vary substantially when genotypes are changed or deleted from the data^21^. Second, each of the locations used in this study may be distinct and cannot be grouped with any other location. This is supported by the lack of repeatable associations among environments. Third, the biplot itself couldn’t capture all of the complex G × E variation and hence the patterns may not be sufficiently clear. A repeatable which-won-where pattern over years is a necessary and sufficient condition for mega-environment delineation^58^. Moreover, this should be based on data in which the same set of genotypes was tested in the same set of test environments across multiple years^33^, which may be facilitated in most of the cases. Also, repeatable associations between locations cannot be relied upon and may not be grouped into different mega-environments due to the dynamic nature of soil salinity, which varies spatially as well as temporally^20, 22^. It is possible to obtain repeatable inferences if we use the same locations and genotypes for testing across years to the extent possible. But, that could be through an academic study but not forward-looking breeding processes, which always try to omit inferior genotypes and add more to test performance. However, if the mega-environment patterns are repeatable, breeders can go ahead with focused breeding efforts and rely more on such mega-environments for better inferences.

## Conclusions

The location CSSRI sodic environment was the most discriminating one and could be used for decisions on genotypes to retain for further testing and release. Overall, the most promising genotypes (CSR-2K-219, CSR-2K-262, and CSR-2K-242) with high mean yield and stability could be used for commercial cultivation across salt-affected soils. The lack of repeatable associations among the locations and repeatable mega-environment groupings suggests the complexity of the abiotic stress soil salinity. This warrants multi-location testing for about five years under salt-affected environments to group locations into distinct mega-environments before deciding about the real value of the location to discriminate and be representative for the target stress. The present study highlights the fact that which-won-where plots could be used to identify the winners but not as the sole criterion for mega-environment grouping. For the time being, continuity of the testing locations for a few more years and improving the efficiency of the less discriminating sites could be a better option for strengthening the results obtained so far. Salinity and alkalinity are two different kinds of stress and they exert stress on plants differently. Genotypes CSR-2K-262, RP 4353-MSC-38-43-6-2-4-3, CSR-2K-219, and NDRK 11-1 were identified as the most promising genotypes under saline soils consistently over years but CSR-2K-262 was the only genotype that was most promising and consistent under alkaline/sodic conditions over three years of testing consistently; hence, it could be suggested for both saline and alkaline conditions. Location-specific ambient conditions contribute to the success of a cultivar; hence, multi-location testing is one of the best ways for identifying genotypes with specific adaptation to a particular agro-climatic zone.

## Methods

### Plant materials and testing locations

**Figure 5:**
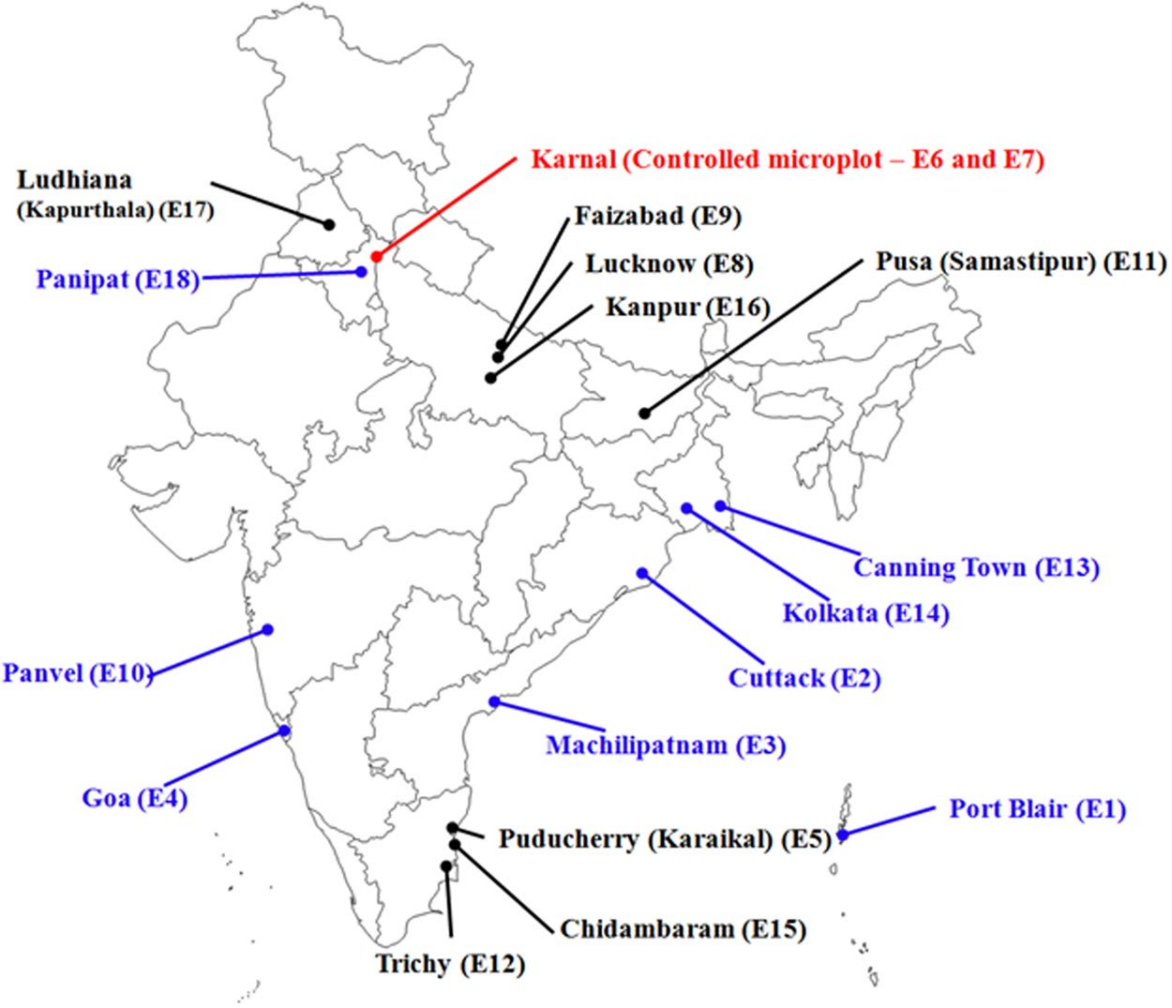
Geographic location of sodic (black color) and saline (blue color) environments in India (modified from www.d-maps.com/carte.php?num_car=24853&lang=en).

The experiment was conducted across 18 locations that comprised nine saline and nine alkaline environments that adequately represent the diverse salt stress conditions in India (Fig. 5). The soils in coastal saline locations varied from sandy loam to clay loam, with ECe (electrical conductivity of saturation extract) of 3.0–17.0 dS m^−1^ and pH of 5.0–7.5 (measured as pH_1:2_, meaning that the pH has a mix of 1 part soil and 2 parts distilled water; hereinafter referred to as pH). Similarly, soils in alkaline locations varied from sandy loam to clay loam, with ECe of 1.0–3.7 dS m^−1^ and pH of 8.3–10.2 (Table 5). Salinity and alkalinity were measured before transplanting and at the time of transplanting, flowering, and maturity. The testing sites were increased each year after the first year of testing with 12, 14, and 18 locations in 2011, 2012, and 2013, respectively. All the experiments were conducted in the wet season.

**Table 5:** Description and characterization of the locations used for the evaluation of rice genotypes during 2011–13 (3 years).

Sl. no.	Location	Biplot code	Years of testing	Latitude & longitude	Stress type	Av. ECe (dS/m)	Av. pH	Mean yield (kg ha^−1^)		
								2011	2012	2013
1	Central Island Agricultural Research Institute (CIARI), Port Blair, Andaman and Nicobar Islands	E1	2011–13	11°37′N	Coastal saline	4.5	5.0	2793	1153	1788
				92°43′E						
2	National Rice Research Institute (NRRI), Cuttack, Odisha	E2	2011	20°27′N	Coastal saline	7.3	5.5	1371	—	—
				85°56′E						
3	Indian Institute of Rice Research (IIRR), Machilipatnam, Andhra Pradesh	E3	2011–13	16°17′N	Coastal saline	8.6	7.5	1804	3449	3448
4	Central Coastal Agricultural Research Institute (CCARI), Ela, Goa	E4	2011–13	15°29′N	Coastal saline	6.2	5.9	1030	2572	1145
				73°55′E						
5	Pandit Jawaharlal Nehru College of Agriculture and Research Institute (PAJANCOA), Karaikal, Puducherry	E5	2011–13	10°55′N	Alkaline	1.9	8.3 (RSC-8)	3042	2658	2412
				79°50′E						
6	Central Soil Salinity Research Institute (CSSRI), Karnal saline microplot, Haryana	E6	2011–13	29°42′N	Inland saline	10.0	7.5	976	1183	1753
				76°57′E						
7	CSSRI, Karnal sodic microplot, Haryana	E7	2011–13	29°42′N	Alkaline	1.4	9.9	539	936	1658
				76°57′E						
8	CSSRI, Regional Research Station, Lucknow, Uttar Pradesh	E8	2011–13	26°86′N	Alkaline	1.1	9.6	576	3441	3574
				80°90′E						
9	Narendra Dev University of Agriculture and Technology (NDUAT), Faizabad, Uttar Pradesh	E9	2011–13	26°77′N	Alkaline	2.8	9.7	2360	4226	2576
				82°15′E						
10	Dr. Balasaheb Sawant Konkan Krishi Vidyapeeth (BSKKV) Kharland Research Station, Panvel	E10	2011–13	18°99′N	Coastal saline	4.0	7.4	2427	2917	2902
				73°12′E						
11	Rajendra Agricultural University (RAU), Pusa, Bihar	E11	2011–13	25°58′N	Alkaline	2.0	9.3	2382	1396	2765
				85°38′E						
12	Anbil Dharmalingam Agricultural College and rch Institute (ADACRI), Trichy, Tamil Nadu	E12	2011–13	10°47′N	Alkaline	1.0	9.1	2581	2746	3550
				78°42′E						
13	CSSRI RRS, Canning Town, West Bengal	E13	2012–13	22°32′N	Coastal saline	3.0	6.7	—	2498	3460
				88°67′E						
14	Centre for Strategic Studies (CSS), Kolkata, West Bengal	E14	2012–13	22°47′N	Coastal saline	9.2	6.20	—	2473	3101
				88°39′E						
15	Annamalai University (AU), Tamil Nadu	E15	2012–13	11°23′N	Alkaline	3.7	8.6 (RSC-7.9)	—	727	1196
				79°43′E						
16	Chandra Shekhar Azad University of Agriculture and Technology (CSAUAT), Kanpur, Uttar Pradesh	E16	2013	26°29′N	Alkaline	1.2	10.2	—	—	1252
				80°18′E						
17	Punjab Agricultural University (PAU), Ludhiana, Punjab	E17	2013	30°53′N	Alkaline	1.0	9.0	—	—	4291
				75°48′E						
18	CSSRI, Nain Farm, Panipat, Haryana	E18	2013	29°19′N	Inland saline	17.0	7.5	—	—	827
				76°47′E						

A total of 53 genotypes were tested during 2011 to 2013 across the selected locations with three national checks, CST 7-1 (coastal saline), CSR 27 (inland saline), and CSR 36 (alkaline). In the first year (2011), 22 salt-tolerant genotypes were evaluated across 12 salt stress locations. Among these, the 10 best genotypes were promoted to the next year of testing (2012). In 2012, 17 new genotypes along with the previous year’s 10 promising genotypes were tested across 14 salt stress locations. Among these 17, the top four genotypes were selected and promoted to the next year. In 2013, the 10 best genotypes from 2011 and 4 genotypes from 2012 were assessed across 17 stress locations along with 14 new entries. The experiment was severely damaged due to high salt stress along with submergence at NRRI, Cuttack (E2). Sowing was done at different sites from the last week of May to the first week of June. The trials were laid out in a randomized complete block design with three replications. The genotypes tested in each year were different as inferior genotypes were discarded and new breeding lines were added every year. A total of 22, 27, and 28 genotypes were tested in 2011, 2012, and 2013, respectively. The details of these genotypes with their pedigrees appear in Table 6. At each site, data on days to 50% flowering, plant height, panicle length, number of filled grains/panicle, 1000-grain weight, and grain yield were recorded. Grain yield was determined from the whole plot and expressed in t/ha.

**Table 6:** Details of rice genotypes used in the study.

**Code**	**Genotype**	**Parentage**	**Years of testing**
G1	RAU-1428-13-7	Sita/Type-3	2011
G2	RAU-1-16-48	EMS mutant of Rasi	2011
G3	CR 2218-64-1-327-4-1	Savitri/Patnai-1	2011
G4	CR 2218-207-3-1-1	Savitri/Patnai-1	2011
G5	CR 2461-1-122-2-1	Gayatri/IET14652	2011
G6	CR 2462-1-154-1-1	Gayatri/Patnai-1	2011
G7	CR 2219-44-2	Savitri/Rahaspunjar	2011
G8	CARI Dhan 2	Milyang 55	2011
G9	CARI Dhan 5	Somaclonal variant of Pokkali	2011
G10	NDRK 11-1	Savita/Annada	2011–13
G11	NDRK 11-2	Selection from traditional landrace Gujarat-70	2011–13
G12	NDRK 11-3	Selection from traditional landrace Amghaur	2011–13
G13	NDRK 11-4	Azucena/IR64	2011–13
G14	NDRK 11-5	BWO 267-3	2011–13
G15	RP 4353-MSC-38-43-6-2-4-3	IET9993/N52	2011–13
G16	RP 4631-146-19-1-1-2-3	CSR3/Kasturi	2011
G17	PNL 9-1-2-7-4-6-1	PNL 1/PNL 3	2011
G18	PNL 1-1-1-6-7-1	IR64/PAK Basmati	2011
G19	CSR-2K-219	Jaya/CSR 23	2011–13
G20	CSR-2K-242	IR5711-3B-7-B-9-1-8/NDRK 5032	2011–13
G21	CSR-2K-255	*O*. *perennis*/CSR 21	2011–13
G22	CSR-2K-262	IR72/CSR 23	2011–13
G23	PNL 9-1-2-7-4-6-1	PNL 1/PNL 3	2012
G24	PNL 1-1-1-6-7-1-1	IR64/PAK Basmati	2012
G25	CR 2815-4-3-1-1-1-1	Annapurna/FL478	2012
G26	CR 2815-4-23-8-5-4-2-1	Annapurna/FL478	2012
G27	CR 2815-4-26-1-S-3-1-1	Annapurna/FL478	2012
G28	CARI Dhan 4	Somaclonal variant of Pokkali	2012
G29	Amal-Mana	Pankaj/SR 26B	2012
G30	CSRC(D)7-0-4	SR 26B/Pankaj	2012
G31	CSR 12-B 18	CSR 23/CSR 27	2012
G32	CSR 12-B 19	CSR 23/CSR 27	2012
G33	CSR 12-B 23	CSR 23/CSR 27	2012–13
G34	NDRK 11-6	IRRI introduction 507 (EC 541934)	2012–13
G35	NDRK 11-7	IR10206-29-2-1/Kuatikputih	2012–13
	(IR65199-4B-10-1-2)		
G36	PNL 4-35-20-4-1-4	IR64/PNL 2	2012–13
G37	CR 2218-41-2-1-1-S-B-1	Savitri/Patnai	2012
G38	CR 2840-1-3-3B-S-B	IR64/FL496	2012
G39	CR 2839-1-S-13-1-5-B-1	Swarna/FL496	2012
G40	NDRK 11-8	BW 267-3	2013
G41	NDRK 11-14	CSRC(s)-2-1-7/N. Usar 3//N. Usar 3	2013
G42	NDRK 11-17	Sarjoo 52/N. Usar 3	2013
G43	KR 09003	Mutant of Improved white ponni	2013
G44	KR 09009	Mutant of Improved white ponni	2013
G45	CSRC(D)2-17-5	Pankaj/NC 678	2013
G46	CSRC(D)12-8-12	Pankaj/Najani	2013
G47	CSRC(D)13-16-9	Pankaj/Gavir Saru	2013
G48	CR 2814-2-4-3-1-1-1	Naveen/FL478	2013
G49	CR 2218-64-1-327-4-1	Savitri/Patnai	2013
G50	CR 2815-4-23-7-5-2-1-1	Annapurna/FL478	2013
G51	CSR 11-121	CSR 11/MI48	2013
G52	CSR 27-192	CSR 27/MI48	2013
G53	CSR 10-M2-27	CSR 10/IR20//IR26	2013
C1	CST 7-1	Coastal saline check	2011–13
C2	CSR 27	Inland saline check	2011–13
C3	CSR 36	Alkaline check	2011–13
LC	Local check	Locally adapted variety	2011–13

### Statistical analysis

Since the locations and genotypes were different between years of testing, analysis was done separately for each year. Additionally, the genotypes common over three years were used for pooled analysis to evaluate their performance over different year and location combinations. Codes were used for locations (Table 1) and genotypes (Table 2) in the individual years 2011, 2012, and 2013. GGE biplot software was used for the option of heritability adjusted GGE (HA-GGE) biplot analysis^27^. The biplots were generated using scaling (2), centering (2), and SVP (2) GGE biplot software. We constructed biplots using those principal components that were having information ratio (IR) > 1. Yield data from a multi-location trial consist of three components: a genotype main effect (G), an environmental component (E), and an interaction term of genotype and environment (GE). But, for the identification of superior cultivars, only G and GE are of utmost importance. A GGE biplot is first constructed by centering the G × E two-way table by environmental means and subjecting the environment-centered data matrix to singular value decomposition (SVD), which yields three component matrices: the SV matrix, the genotype eigenvector matrix, and the environment eigenvector matrix.

The general model for GGE biplots is as follows:

1 $${p}_{ij}=\frac{{\bar{y}}_{ij}-\,{\mu }_{j}}{{S}_{j}}=\sum _{k=1}^{t}{\lambda }_{k}{\alpha }_{ik}{\gamma }_{jk}+{\bar{\varepsilon }}_{ij}$$

Here, the GGE biplot used is heritability adjusted (HA) and hence the scaling factor *S*_*j*_ becomes SD/$\sqrt{H}$. The cosine of angle (α_jj’_) between two environments is measured as genetic correlation between them^10, 11, 16^

2 $${r}_{g(jj^{\prime} )}=\,\cos \,{\alpha }_{jj^{\prime} }$$

The genetic gain (G) observed in target environment j’ through indirect selection in test environment j is given by ref. 26

3 $$G={i}_{j}\,({h}_{j}{r}_{g(jj^{\prime} )})\,{\sigma }_{g(j^{\prime} )}$$

The discriminating ability of a location is judged by the length of its vector. Further, desirability index of the locations across years were calculated as product of representativeness and discriminating ability estimates. The projection of a genotype from the average tester (AT) axis is used to decide the stability of the genotype. The “which-won-where” option was used to identify which genotype was the winner in a given set of environments and to categorize mega-environments. The biplots were interpreted as described by Yan^17^.

## Electronic supplementary material

Supplementary tables
