# Supplementary material for: Identification of mega-environments and rice genotypes for general and specific adaptation to saline and alkaline stresses in India
Source: Sci Rep. 2017 Aug 11;7:7968. doi: 10.1038/s41598-017-08532-7 (PMC5554213; doi:10.1038/s41598-017-08532-7)
Supplement: Supplementary file 1 — Supplementary tables [file 41598_2017_8532_MOESM1_ESM.doc]

**Identification of mega-environments and rice genotypes for general and specific adaptation to saline and alkaline stresses in India**

S. L. Krishnamurthy^1*^, P. C. Sharma^1^, D. K. Sharma^1^, K. T. Ravikiran^1^, Y. P. Singh^2^, V. K. Mishra^2^, D. Burman^3^, B. Maji^3^, S. Mandal^3^, S. K. Sarangi^3^, R. K. Gautam^4^, P.K. Singh^4^, K. K. Manohara^5^, B. C. Marandi^6^, G. Padmavathi^7^, P. B. Vanve^8^, K. D. Patil^8^, S. Thirumeni^9^, O. P. Verma^10^, A. H. Khan^10^, S. Tiwari^11^, S. Geetha^12^, M. Shakila^12^, R Gill^13^, V. K. Yadav^14^, S. K. B. Roy^15^, M. Prakash^16^, J. Bonifacio^17^, Abdelbagi Ismail^18^, G. B. Gregorio^17^, Rakesh Kumar Singh^17*^

^1^Central Soil Salinity Research Institute, Karnal, India.

^2^Central Soil Salinity Research Institute, Regional Research Station, Lucknow, India.

^3^Central Soil Salinity Research Institute, Regional Research Station, Canning Town, India.

^4^Central Island Agricultural Research Institute, Port Blair, A & N Islands, India.

^5^Central Coastal Agricultural Research Institute (CCARI), Ela, Goa, India.

^6^National Rice Research Institute (NRRI), Cuttack, Odisha, India.

^7^Indian Institute of Rice Research, Telengana, India.

^8^Dr. Balasaheb Sawant Konkan KrishiVidyapeeth, Khar Land, Panvel, India.

^9^Pandit Jawaharlal Nehru College of Agriculture and Research Institute, Karaikal, India.

^10^Narendra Deva University of Agriculture & Technology, Faizabad, Uttar Pradesh, India.

^11^Rajendra Agricultural University, Samastipur, India.

^12^Anbil Dharmalingam Agricultural College and Research Institute, Trichy, India.

^13^ Punjab Agricultural University, Ludhiana, India.

^14^Chandra Shekhar Azad University of Agriculture & Technology, Kanpur, Uttar Pradesh, India.

^15^Centre for Strategic Studies, Salt Lake City, India.

^16^Annamalai University, Chidambaram, Tamil Nadu, India.

^17^Division of Plant Breeding, IRRI, Philippines.

^18^Crop and Environmental Sciences Division, IRRI, Philippines.

* Corresponding authors:

S. L. Krishnamurthy: [krishnagene@gmail.com](mailto:krishnagene@gmail.com)

Rakesh Kumar Singh: [r.k.singh@irri.org](mailto:r.k.singh@irri.org)

**Supplementary table 1. Summary statistical estimates for 18 locations in individual years (2011, 2012, and 2013)**

| **Location** | **2011** |  |  |  | **2012** |  |  |  | **2013** |  |  |  |
| --- | --- | --- | --- | --- | --- | --- | --- | --- | --- | --- | --- | --- |
|  | Mean | Max | SE | SD | Mean | Max | SE | SD | Mean | Max | SE | SD |
| E1 | 2872.05 | 5729.17 | 540.76 | 1658.786 | 1171.70 | 3000.00 | 269.80 | 848.22 | 1790.97 | 2346.67 | 192.43 | 312.59 |
| E2 | 1456.75 | 2608.10 | 255.69 | 500.784 | - |  | - | - | - |  | - | - |
| E3 | 1789.08 | 2671.50 | 189.45 | 363.405 | 3436.53 | 4671.00 | 521.41 | 867.40 | 3426.40 | 4671.00 | 515.58 | 838.03 |
| E4 | 1117.43 | 1827.58 | 170.42 | 521.552 | 2588.05 | 3620.83 | 433.16 | 613.69 | 1149.02 | 2419.17 | 367.18 | 550.92 |
| E5 | 3018.45 | 5061.11 | 511.21 | 1122.066 | 2631.91 | 4138.67 | 337.73 | 666.24 | 2411.20 | 3626.33 | 279.45 | 618.03 |
| E6 | 958.49 | 1830.07 | 84.39 | 452.984 | 1165.56 | 2614.55 | 80.79 | 653.41 | 2107.77 | 2991.67 | 11.95 | 439.65 |
| E7 | 522.53 | 992.49 | 62.66 | 261.640 | 896.45 | 2570.11 | 72.66 | 623.13 | 1948.67 | 2602.33 | 7.62 | 394.99 |
| E8 | 753.37 | 1568.60 | 656.41 | 439.244 | 4266.96 | 6238.10 | 1008.23 | 1309.79 | 4414.80 | 6000.48 | 587.81 | 770.86 |
| E9 | 2655.11 | 4315.33 | 192.16 | 1118.585 | 4839.51 | 6515.33 | 273.74 | 1133.49 | 2938.11 | 3734.06 | 212.36 | 406.70 |
| E10 | 2386.91 | 4268.67 | 50.26 | 1099.107 | 3102.12 | 3775.00 | 59.04 | 366.82 | 2867.30 | 6257.67 | 150.57 | 1459.07 |
| E11 | 2392.38 | 3000.00 | 576.86 | 351.081 | 1402.67 | 2333.33 | 268.23 | 413.58 | 2740.65 | 3653.33 | 586.34 | 566.89 |
| E12 | 2561.92 | 6868.67 | 102.69 | 1633.774 | 2771.29 | 6383.33 | 125.32 | 1187.34 | 3774.13 | 5317.59 | 751.89 | 1084.60 |
| E13 |  |  |  |  | 2468.29 | 4001.39 | 450.27 | 883.67 | 3412.20 | 4400.00 | 909.03 | 494.25 |
| E14 |  |  |  |  | 2653.91 | 3933.33 | 411.00 | 515.36 | 3709.62 | 5333.33 | 670.86 | 985.66 |
| E15 |  |  |  |  | 731.70 | 1673.00 | 3.72 | 364.06 | 1180.34 | 1864.69 | 49.15 | 258.93 |
| E16 |  |  |  |  |  |  |  |  | 1257.48 | 2770.00 | 133.25 | 683.09 |
| E17 |  |  |  |  |  |  |  |  | 4868.08 | 6565.84 | 798.71 | 811.53 |
| E18 |  |  |  |  |  |  |  |  | 1203.67 | 2527.33 | 33.54 | 572.26 |

**Supplementary table 2.** Summary of discriminating, representative, and ideal locations in different seasons (2011-13)

| **Year** | **Discriminating** | **Representative** | **Ideal** |
| --- | --- | --- | --- |
| 2011 | CSSRI, Karnal saline environment (E6) | ADACRI, Trichy (E12) | ADACRI, Trichy (E12) |
| 2012 | CSSRI Karnal sodic environment (E7) | CSSRI RRS, Lucknow (E8), CSSRI Karnal sodic environment (E7), NDUAT, Faizabad (E9) | CSSRI Karnal sodic environment (E7) |
| 2013 | CSSRI Karnal sodic environment (E7) | NDUAT, Faizabad (E9) | PAU, Ludhiana (E17) |
| 2011-13 | CSSRI Karnal sodic environment (E7_13) | CSSRI RRS, Lucknow (E8_2012) | NDUAT, Faizabad (E9_2012) |

**Supplementary table 3. Mega-environments and their corresponding winning genotypes for grain yield over different years (2011-13)**

| **Year** | **Winning genotype** | **Mega-environment** | **No. of locations** |
| --- | --- | --- | --- |
| 2011 | CSR 36 (C3) | IIRR, Hyderabad (E3); CSSRI, Karnal saline environment (E6); CSSRI, Karnal sodic environment (E7); and CSSRI RRS, Lucknow (E8) | 4 |
|  | CSR 27 (C2) | CCARI, Goa (E4); BSKKV, Kharland RS, Panvel (E10); RAU, Pusa, Bihar (E11); and ADACRI, Trichy (E12) | 4 |
|  | NDRK 11-2 (G11) | CIARI, Port Blair (E1); PAJANCOA, Karaikal, Puducherry (E5); and NDUAT, Faizabad (E9) | 3 |
| 2012 | Amalmana (E29) | IIRR, Machilipatnam (E3); CSSRI RRS, Canning Town (E13); and RAU, Pusa, Bihar (E11) | 3 |
|  | CSR2K-262 (G22) | CSSRI, Karnal saline environment (E6); Kharland RS, Panvel (E10); CIARI, Port Blair (E1); Annamalai University (E15); CCARI, Goa (E4); and NDUAT, Faizabad (E9) | 6 |
|  | CR 2815-4-3-1-1-1-1 (G26) | ADACRI, Trichy (E12) and CSS, Kolkata (E14) | 2 |
| 2013 | CSRC(D)12-8-12 (G46) and CSRC(D)13-16-9 (G47) | CSSRI, Karnal saline environment (E6); CSSRI, Karnal sodic environment (E7); CSSRI RRS, Lucknow (E8); NDUAT, Faizabad (E9); CSSRI RRS, Canning Town (E13); PAU, Ludhiana (E17); and CSSRI, Nain Farm, Panipat (E18) | 7 |
|  | CR 2814-2-4-3-1-1-1 (G48) and CR 2815-4-23-7-5-2-1-1 (G50) | ADACRI, Trichy (E12); CSAUAT, Kanpur (E16); PAJANCOA, Karaikal, Puducherry (E5); and CCARI, Goa (E4) | 4 |
|  | CST 7-1 (C1) and RP 4353-MSC-38-43-6-2-4-3 (G15) as winning genotypes | CIARI, Port Blair (E1); RAU, Pusa, Bihar (E11); BSKKV, Kharland RS, Panvel (E10); and CSS, Kolkata (E14) | 4 |
| 2011-13 | CSR2K-262 (G22) | CSSRI RRS, Lucknow_11; Karnal sodic environment_12; CSSRI, Nain Farm_13; Karnal sodic environment_11; CSSRI, Karnal saline environment_13; CSSRI, Karnal sodic environment_13; PAU, Ludhiana_13; NDUAT, Faizabad_13; ADACRI, Trichy_12; BSKKV, Kharland RS, Panvel_12; ADACRI, Trichy _11; CSSRI, Karnal saline environment _11; CSSRI RRS, Lucknow_13; CSSRI RRS, Lucknow_12; CCARI, Goa _12; Annamali University_13 | 16 |
|  | NDRK 11-3 (G12), CSR 36 (C3) | RAU, Pusa, Bihar_11; ADACRI, Trichy_13; NRRI, Cuttack_11; CIARI, Port Blair_12; NDUAT, Faizabad_12; Annamalai University_12; CCARI, Goa _11; BSKKV, Kharland RS, Panvel_11; IIRR, Machilipatnam _11 | 9 |
|  | RP 4353-MSC-38-43-6-2-4-3 (G15) | BSKKV, Kharland RS, Panvel_13; CSS, Kolkata_13; CIARI, Port Blair_2013; CSSRI RRS, Canning Town_12; IIRR, Machilipatnam_12; IIRR, Machilipatnam_13; RAU, Pusa, Bihar_12; CSSRI RRS, Canning Town_13 | 8 |
|  | CSR-2K-255 (G21) | CCARI, Goa_13; NDUAT, Faizabad_11; RAU, Pusa, Bihar_13; CIARI, Port Blair_11; PAJANCOA, Karaikal, Puducherry_11; PAJANCOA, Karaikal, Puducherry_12; PAJANCOA, Karaikal, Puducherry_13; CSAUAT, Kanpur_13; CSS, Kolkata_12; Karnal saline environment_12 | 10 |

**Supplementary table 4**. Genotypes with the highest mean yield across years specific to saline and sodic conditions

| **Saline** | **Sodic** |
| --- | --- |
| CSR-2K-262 (2845)  RP 4353-MSC-38-43-6-2-4-3 (2410)  CSR-2K-219 (2353)  NDRK 11-1 (2285) | CSR-2K-262 (3092) |

*Numbers in parentheses indicate grain yield (kg ha^-1^) pooled across locations and years.
